# Genome-wide analysis of UDP-glycosyltransferase gene family and identification of a flavonoid 7-*O-*UGT (*AhUGT75A*) enhancing abiotic stress in peanut (*Arachis hypogaea* L.)

**DOI:** 10.1186/s12870-023-04656-3

**Published:** 2023-12-07

**Authors:** Lei Ouyang, Yue Liu, Ruonan Yao, Dongli He, Liying Yan, Yuning Chen, Dongxin Huai, Zhihui Wang, Bolun Yu, Yanping Kang, Huifang Jiang, Yong Lei, Boshou Liao, Xin Wang

**Affiliations:** 1 Key Laboratory of Biology and Genetic Improvement of Oil Crops, Ministry of Agriculture and Rural Affairs, Oil Crops Research Institute of the Chinese Academy of Agricultural Sciences, Wuhan, 430062 P.R. China; 2 State Key Laboratory of Biocatalysis and Enzyme Engineering, School of Life Sciences, Hubei University, Wuhan, 430062 P.R. China

**Keywords:** UDP-glycosyltransferase, Expression analysis, Peanut, Abiotic stress, Flavonoid

## Abstract

**Background:**

Glycosylation, catalyzed by UDP-glycosyltransferase (UGT), was important for enhancing solubility, bioactivity, and diversity of flavonoids. Peanut (*Arachis hypogaea* L.) is an important oilseed and cash crop worldwide. In addition to provide high quality of edible oils and proteins, peanut seeds contain a rich source of flavonoid glycosides that benefit human health. However, information of UGT gene family was quite limited in peanut.

**Results:**

In present study, a total of 267 *AhUGTs* clustered into 15 phylogenetic groups were identified in peanut genome. Group I has greatly expanded to contain the largest number of *AhUGT* genes. Segmental duplication was the major driving force for *AhUGT* gene family expansion. Transcriptomic analysis of gene expression profiles in various tissues and under different abiotic stress treatments indicated *AhUGTs* were involved in peanut growth and abiotic stress response. *AhUGT75A* (*UGT73CG33*), located in mitochondria, was characterized as a flavonoid 7-*O-*UGT by in vitro enzyme assays. The transcript level of *AhUGT75A* was strongly induced by abiotic stress. Overexpression of *AhUGT75A* resulted in accumulating less amount of malondialdehyde (MDA) and superoxide, and enhancing tolerance against drought and/or salt stress in transgenic *Arabidopsis*. These results indicated *AhUGT75A* played important roles in conferring abiotic stress tolerance through reactive oxygen species scavenging.

**Conclusions:**

Our research only not provides valuable information for functional characterization of UGTs in peanut, but also gives new insights into potential applications in breeding new cultivars with both desirable stress tolerance and health benefits.

**Supplementary Information:**

The online version contains supplementary material available at 10.1186/s12870-023-04656-3.

## Background

The current global environmental degradation severely affects plant growth and development. Abiotic stresses, including drought, cold and salinity, give rise to excessive production of reactive oxygen species (ROS) in plants and disruption of cellular membrane and ionic equilibriums, thus leading to decrease in crop yields and severe economic losses each year [1, 2]. Plants have evolved various secondary metabolites to mitigate the damage caused by stressful conditions in order to survive [3]. Flavonoids, exist mostly in the form of glucosides *in planta*, play vital roles in maintaining redox homeostasis and conferring abiotic stress tolerance [4].

Glycosylation usually occurs in the final step of flavonoid biosynthesis, and this modification can enhance compound solubility, stability, bioactivity, and diversity [5]. The reaction of glycosylation was catalyzed by family 1 glycosyltransferases, also named uridine diphosphate glycosyltransferases (UGTs), which transfer a glycosyl group from UDP-sugars to a board range of acceptors, including plant hormones, secondary metabolites, and xenobiotics [6]. Plant UGTs contained a conserved motif of 44 amino acids closed to their C-terminal, termed plant secondary product glycosyltransferase (PSPG) box, which was responsible for the UDP-sugar binding site of the enzymes [7]. In model plant *Arabidopsis thaliana*, there are a total of 123 putative UGTs, which could be classified into 14 groups (A-N) according to phylogenetic analysis [8]. With the rapid development of bioinformatics and genomics, many UGT gene family members have been identified in other plant species, with 147 in *Zea mays* [9], 149 in *Glycine max* [10], 409 in *Medicago sativa* [11], 184 in *Oryza sativa* [8], and 274 in *Gossypium hirsutum* [12]. Subsequently, four novel groups (O, P, Q, R), which are not presented in *Arabidopsis*, have been discovered based on their phylogenetic relationships [8].

The functions of plant UGTs have been reported to be involved in growth and stress responses [13, 14]. A subset of *UGT* transcript levels were found to be enhanced by various abiotic stimuli, such as cold, salt, and drought [15, 16]. In *Arabidopsis*, two stress-induced *UGTs* (*UGT79B2* and *UGT79B3*) were characterized as anthocyanin rhamnosyltransferases, and conferred plant adaptation to abiotic stresses via regulating anthocyanin metabolism [17]. In tea plants, three *UGTs* (*CsUGT91Q2*, *CsUGT78A14* and *CsUGT78A15*) were involved in cold tolerance by glycosylation of sesquiterpenes and flavonols [18–20]. Group D was one of the largest subgroups of plant UGT family. Several UGT73 members were found to be scattered in this subgroup, which could recognize a variety of substrates including terpenoids and flavonoids [21–23]. In soybean, the expression level of *GmUGT73F4* (belonging to group D) was strong induced by high temperature and humidity. Heterogenous overexpression of *GmUGT73F4* in *Arabidopsis* improved seed viability and abiotic stresses tolerance by up-regulating stress-related genes and increasing ROS scavenging capability [24].

Peanut (*Arachis hypogaea* L., also called groundnut) is an important oilseed and cash crop worldwide. In addition to provide high quality of edible oils and proteins, peanut seeds contain a rich source of flavonoid glycosides that benefit human health [25, 26]. As a typical thermophilic crop, peanut requires relatively warm temperatures throughout the whole developmental stages [27]. The growth of peanut is severely inhibited below 15^o^C, and cold stress (non-freezing) is deemed as a limiting factor in peanut cultivation and production [28]. Moreover, despite being a rainfed crop, peanut is sensitive to drought stress during the flowering and pegging stages. It has been reported that higher drought-tolerant cultivars generally contained more flavonoid contents, suggesting these compounds were likely to be involved in defense against drought stress in peanut [29]. Since UGTs were the key enzymes in flavonoid biosynthesis, it was proposal that they play vital roles in peanut stress responses. However, the information of peanut UGT gene family is very limited, and no UGT has been functionally characterized in peanut so far. In present study, a total of 267 *AhUGT* members were identified from peanut genome. Their phylogenetic relationships, chromosome distributions, gene structures and expression patterns were comprehensively analyzed. *AhUTG75A* was characterized as a flavonoid 7-*O-*glucosyltransferase by in vitro enzyme assays. Overexpression of *AhUTG75A* enhances drought and salt stress tolerance in transgenic *Arabidopsis*. This study will not only facilitate to functional characterization of *AhUGTs*, but also provide new insights into their potential applications in breeding peanut cultivars with both desirable stress tolerance and health benefits.

## Materials and methods

### Plant materials, growth condition and stress treatment

*Arachis hypogaea* cv. Zhonghua-215 is an elite variety released by Oil Crops Research Institute Chinese Academy of Agricultural Sciences. Zhonghua-215 cultivar was selected for this experiment, since it has a good comprehensive performance, with high and stable yields as well as strong lodging-resistance. Moreover, this cultivar is moderately tolerant to different stressful conditions, including salt, cold and drought. Peanut seeds were soaked in water for six hours, and put into petri dishes with moistened filter papers for germination. Three-day-old uniform seedlings were transferred into plastic cuboid container with Hoagland nutrient solution in a greenhouse (16 h/8 h light/dark cycles at 28 ^o^C). After 14 days, seedlings were exposed to low temperature (10 ^o^C) for cold stress. 150 mM NaCl or 20% polyethylene glycol 6000 (PEG-6000) solutions were applied for salt or drought treatment, respectively. The control condition (CK) was created using sterile water. After 0, 3, 6, 12, 24, 48 h treatment, roots and the third unfolded leaves were cut from the seedlings, and all samples were immediately frozen in liquid nitrogen and then stored at -80 °C until use.

### Identification of UGTs in peanut

To identify members of UGT gene family in peanut, *A. hypogaea* cv. Tifrunner genome information was downloaded from peanutbase (https://www.peanutbase.org). The hidden Markov model (HMM) profile of PF00201.20 (a conserved domain of UGT protein) downloaded from the Pfam website (http://pfam.xfam.org) was used as a query to search for candidate UGTs in *A. hypogaea* genome using HMMER v3.1 software (with a cut off E-value of 1 × 10^− 5^). The reductant and/or incomplete (the length of protein sequence less than 200 amino acids) UGT sequences were removed. Furthermore, the conserved domain of plant secondary product glycosyltransferase (PSPG) box of UGTs was verified by SMART (http://smart.embl.de) and NCBI CDD (https://www.ncbi.nlm.nih.gov/cdd). Molecular weight (MW) and isoelectric point (PI) of UGT proteins were calculated using the online ExPASy program (https://prosite.expasy.org). The subcellular localization was predicted with the CELLO server (http://cello.life.nctu.edu.tw).

### Multiple sequence alignment and phylogenetic analysis

The putative amino acid sequences of UGTs from peanut and other model species were aligned with MAFFT v7.490 with default settings [30]. A phylogenetic tree was constructed by IQ-tree program (v2.2.0) using the maximum likelihood (ML) method with a bootstrap value of 1000 [31]. The tree was visualized with FigTree software (v1.4.4) (http://tree.bio.ed.ac.uk/).

### Chromosomal location and gene duplication

The chromosomal location information of *AhUGTs* was retrieved from the GFF3 file in peanutbase website. MapChart software (v2.32) was used to visualize the chromosomal distribution of *AhUGTs* [32]. The tandem repeat and segmental duplication events of *UGT* genes in the genome were searched using multiple collinear scanning toolkits (MCScanX) with an E-value set to 10^− 5^ [33]. The genome files of *A. duranensis* and *A. ipaensis* were obtained from peanutbase. *A. thaliana* sequence files were retrieved from TAIR 10 (http://www.arabidopsis.org/). The genome data of *Glycine max* and *Gossypium hirsutum* were downloaded from PlantGDB (http://www.plantgdb.org) and CottonGen (https://www.cottongen.org) respectively [34, 35]. The syntenic relationships between *AhUGTs* and *UGT* genes from other species (*A. duranensis*, *A. ipaensis*, *A. thaliana*, *G. max*, and *G. hirsutum*) were mapped using MCScanX [33]. Synonymous (Ks) and non-synonymous substitutions (Ka) of orthologous *AhUGTs* were calculated by TBtools software [36]. The evolutionary time of duplicated pairs was estimated as: T = Ks/2λ (λ = 8.12 × 10^− 9^) [37].

### Analysis of gene structure and conserved motifs

The exon-intron structures were mapped by Gene Structure Display Server (GSDS, http://gsds.gao-lab.org) based on the intron-exon data in the peanut GFF3 annotation file [38]. The conserved motifs of AhUGT proteins were analyzed by motif-based sequence analysis tools (MEME, https://meme-suite.org) [39]. The parameters were set as follows: search time = 18000s, maximum number of motifs = 12, and optimum motif width ranged from 6 to 100 bp. The phylogenetic tree, gene structure and motif were combined and displayed using TBtools software [36].

### Cis-acting element analysis and gene expression pattern

DNA sequences in the 1.5 kb upstream region of *AhUGT* gene transcription start site were obtained from peanut genomic data. The online website PlantCARE (http://bioinformatics.psb.ugent.be/webtools/plantcare/) was used to predict the *cis-*acting elements in the promoter regions of *AhUGTs* [40]. RNA-seq data were downloaded from the peanutbase to investigate the expression pattern of *AhUGTs* in 22 different tissues and developmental stages [41]. To analyze the transcriptional response of *AhUGTs* in peanut seedlings subjected to cold treatment, transcriptome data (accession: PRJNA751249) were downloaded from the NCBI database (https://www.ncbi.nlm.nih.gov/) [42]. Furthermore, expression profiling of *AhUGTs* under cold and/or drought stress was obtained from Peanut Genome Resource (http://peanutgr.fafu.edu.cn) [43]. The gene expression levels were estimated by FPKM values (fragments per kilobase of transcript per million fragments mapped), which were directly obtained from the specific databases. Since the FPKM values of some *AhUGTs* (e.g. *AhUGT53A*, *AhUGT95A*, *AhUGT122B* and *AhUGT149*) varied greatly (ranging from 0 to 471.09) in response to stress treatment, it is difficult to distinguish their differential expression patterns by the gradient color from green to red in the heatmap when using FPKM values. Moreover, if FPKM values was zero, it was unable to calculate log_2_FPKM, therefore, FPKM values were converted to log_2_(FPKM + 1) to perform heatmap analysis. The heatmap of *AhUGT* expression was visualized using R software package “pheatmap”.

### qRT-PCR analysis of *AhUGTs*

Total RNA was extracted from the leaves and roots using Prep Pure Plant Plus kit (Tiangen Biotech Co. Ltd., Beijing, China). After removal of residual genomic DNA with DNase I, about 1 μg of RNA from each sample was reverse-transcribed into cDNA using the M-MLV reverse transcriptase Kit (Thermo Fisher Scientific, USA). qRT-PCR reactions were performed on a CFX96 Real-Time PCR Detection System using SYBR Green qRT-PCR Mix (Sangon, Shanghai, China) as previously reported [44]. qRT-PCR primers were listed in Table S1. Actin gene (accession number: *Aradu.W2Y55*) was used as an internal reference gene.

### Subcellular localization analysis

The open reading frame (ORF) of *AhUGT75A* was amplified by reverse transcription PCR (RT-PCR) with the primers listed in Table S1, and cloned into PC1300-35S::GFP vector. The recombined PC1300-35S::*AhUGT75A*-GFP vector was transformed into *Arabidopsis* protoplasts expressing a red fluorescent mitochondrion marker (mitochondrion-RFP) using PEG-mediated method [45]. After 48 h, the signals of green fluorescent protein (GFP), red fluorescent protein (RFP), and chloroplast auto-fluorescence were observed using Zeiss LSM710NLO confocal laser scanning microscope.

### Heterologous expression and in vitro enzyme assay

The coding sequence of *AhUGT75A* was amplified by RT-PCR with the primers listed in Table S1, and cloned into pGEX-4T vector (GE Healthcare) to give a fusion protein with glutathione-S-transferase (GST) tag. The resulting vector was transformed into *Escherichia coli* BL21 strain for protein expression. The recombinant AhUGT75A protein was extracted and purified using a Glutathione Sepharose 4B kit (GE Healthcare) according to the manufacturer’s manual. The enzymatic activity assays were conducted according to Wang et al. (2016) [46]. The reaction mixtures were incubated at 30 °C for 30 min, and were stopped by adding the same volume of UDP-Glo™ assay reagent [47]. To determine the kinetic parameters for naringenin and resveratrol, eight different substrate concentrations ranging from 8 to 500 μM were applied, V_max_ and K_m_ values were calculated by nonlinear regression analysis.

The reaction products were analyzed on an Agilent 1290 Infinity II HPLC system (Agilent Technologies) equipped with a Luna C18(2) reverse-phase column and separated in a mobile phase consisting of solvent A (1% [v/v] phosphoric acid in Milli-Q water) and solvent B (acetonitrile). Liquid chromatography-mass spectrometry (LC-MS) analysis was performed on an Accela LC system coupled with TSQ Quantum Access Max mass spectrometer (Thermo Scientific, USA). The column and analysis method were same with the HPLC analyses as described above. The MS data were recorded with ranges of *m/z* 100–800.

### Arabidopsis transformation and stress treatment

The coding sequence of *AhUGT75A* was amplified by RT-PCR with the primers listed in Table S1, and cloned into pCAMBIA1300 binary vector under CaMV 35S promoter. The resulting construct was transformed into *Arabidopsis* Col-0 by *Agrobacterium*-mediated floral dip method [48]. The T1 to T3 transgenic lines were verified by PCR using the primers listed in Table S1, and three transgenic lines with differing *AhUGT75A* transcript level were selected for analysis. For stress treatments, one-week-old transgenic seedlings growing on MS medium supplemented with hygromycin were transplanted into soil, and grown for two further weeks with normal irrigation, and then the 3-week-old plants were photographed and harvested as control materials (CK). After that, for drought stress treatment, the plants were left without watering for 17 days, followed by re-irrigation for recovery for 3 days. For salt stress treatment, the three-week-old seedlings were irrigated with 200 mM NaCl solution instead of water for every 3 days, then the performances of the plants were photographed and the leaves were harvested at five and seven days after irrigation. Electrolyte leakage assay was performed according to the protocol described by Shi et al. (2020) [49]. The malondialdehyde (MDA) contents were measured according to thiobarbituric acid (TBA) reaction method by using MDA assay kit (Solarbio Science & Technology Co. Ltd., Beijing, China). Nitrobluetetrazolium (NBT) staining was performed by incubated leaves with 1 mM NBT in 10 mM Tris-HCl buffer (pH 7) at room temperature for 12 h. After straining, leaves were washed three times with ddH_2_O and decolorized in 95% ethanol overnight.

## Results

### Identification and phylogenetic analysis of UGT gene family in *A. hypogaea*

To identify UGT gene family members, the HMM file of PF00201 was used as a query to search against *A. hypogaea* genome. After filtering out redundant and incomplete UGT sequences, a total of 267 putative *UGTs* were obtained from *A. hypogaea* genome. As peanut is an allotetraploid plant (AABB), some of the *UGT* genes in the peanut genome exhibit high similarity and identity. If the AhUGTs shared amino acids sequence identities with more than 95%, they will be named with the same number, using A and B to differentiate them. As a result, the 267 *AhUGTs* were name *AhUGT1A-AhUGT196* based on their chromosomal locations and sequence identities (Table S2). All AhUGT proteins were confirmed by the presence of a conserved PSPG domain. The deduced protein length of AhUGTs ranged from 231 to 903 amino acids (average length 473 aa), and the predicted molecular weight (MW) varied from 25.62 (AhUGT29) to 101.33 kDa (AhUGT58B) with a mean value of 53.17 kDa. The isoelectric point (PI) ranged from 4.66 (AhUGT191) to 8.78 (AhUGT103B) with an average value of 6.29. The subcellular localization analysis showed that most of AhUGTs were predicted to localize in cytoplasm (54) and/or plasma membrane (57), while the remaining were putatively distributed in extracellular matrix (24), chloroplast (17), mitochondria (9), nuclear (5) and lysosome (1) respectively.

**Fig. 1:**
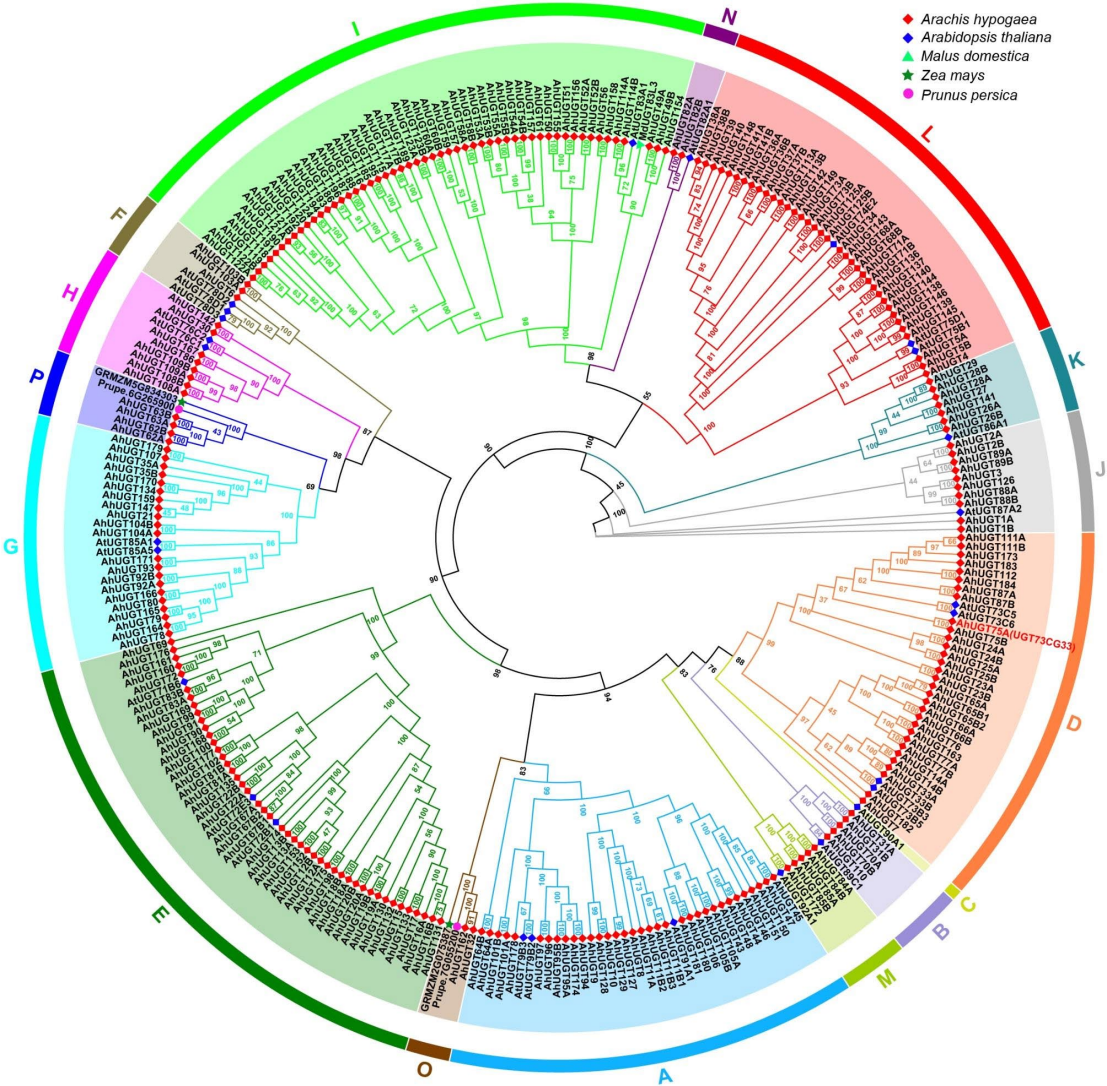
Phylogenetic analysis of *AhUGTs* in *A. hypogaea.* The phylogenetic tree was constructed using the maximum likelihood method by aligning the amino acid sequences of 267 peanut UGTs with those of functionally characterized UGTs from other plant species. The tree was visualized using the FigTree v1.4.4 program. The UGT members are clustered into 16 groups (A–N, and O, P), and indicated by different colors. Bootstrap values are displayed on the nodes

To clarify the evolutionary relationship of *AhUGTs*, a phylogenetic tree was constructed by aligning amino acid sequences of 267 *AhUGTs* with functionally characterized *UGTs* from *Arabidopsis* and other plant species (Fig. 1, Table S3). It has been reported that plant UGTs could be clustered into 18 distinct groups including 14 identified groups in *Arabidopsis* (A to N) as well as the four new groups (O-R) in maize and other plants [8]. According to the tree, 267 *AhUGTs* were scattered into 15 major groups, with the lack of members from group C, Q and R. The expansion/contraction ratios were calculated to further access the relative expansion and contraction rates of *AhUGT* groups [8] (Table S4-5). An over-representation of eight groups (D-J, and L) was observed in *A. hypogaea*, with their expansion ratios more than 1. There were 53 *AhUGT* members in Group I (19.9% of total *AhUGTs*), which had the highest expansion ratio (8.63) among all the groups, indicating group I expanded largely during the evolutionary history of cultivated peanut. On the other hand, five groups (K, M-P) were under represented (with contraction ratios less than 0.5), the contraction of *UGTs* in these groups was also observed in other vascular plants, including *Arabidopsis*, rice, and *M. truncatula* [8].

### Gene structure and conserved sequence analysis

To investigate characteristics of *AhUGT* gene family, their exon-intron structures and conserved motifs were examined (Fig. S1). It was found that 71 *AhUGT* genes (accounting for 27%) contained no introns. About half of *AhUGTs* (138) had one intron, and the remaining 58 members (21%) possessed multiple introns (ranging from two to five). Among of them, the genomic length of *AhUGT86* (12,573 bp, in group H) was longest, followed by *AhUGT52A* (11,291 bp, in group I), which was due to the existence of a large-sized intron within the gene body. Furthermore, a total of 12 conserved motifs were predicated in AhUGT proteins based on MEME analysis. Motif 7 was the consensus PSPG-box present at the C-terminal of all AhUGTs. While motif 8, 9, 10, 11, and 12 occurred in most of AhUGTs, other motifs existed in specific groups. For instance, motif 3 was only found in group D, B and J, and motif 5 was uniquely present in group E and J. It should be noted that *AhUGTs* clustered in the same group displayed different gene structural patterns, but usually shared similar protein motifs.

### Chromosomal distribution and synteny analysis of *AhUGT* genes

**Fig. 2:**
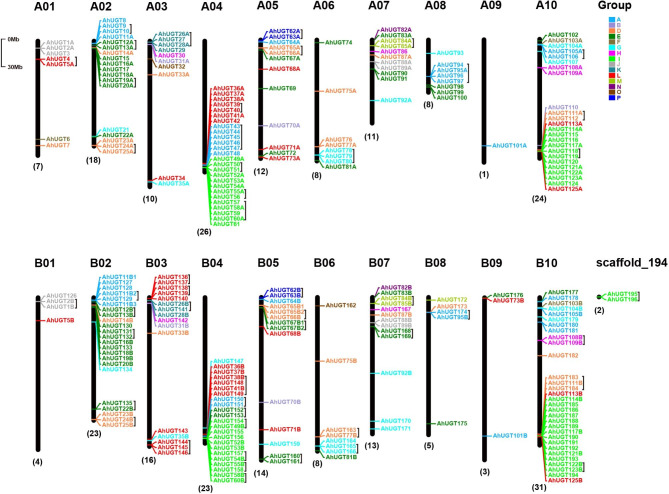
Chromosomal locations and tandem duplication of UGT family in *A. hypogaea*. The counts of *AhUGTs* are indicated at the bottom of each chromosome. Tandem duplicated *AhUGT* genes are enclosed in black lines. The left scale represents a 30-Mb chromosomal distance. *AhUGT* members belonging to distinct phylogenetic groups are indicated by different colors

The chromosomal location of *AhUGT* genes was investigated based on genome annotation retrieved from the peanut database. Among them, 265 *AhUGT* genes (*AhUGT1A-AhUGT194*) were unevenly distributed across 20 chromosomes, while two *UGTs* (*AhUGT195* and *AhUGT196*) were mapped on the scaffolds_194 (Fig. 2). Specifically, B10 has the largest number of *AhUGTs* (31), followed by A04 with 26 members, while only one gene was on A09.

**Fig. 3:**
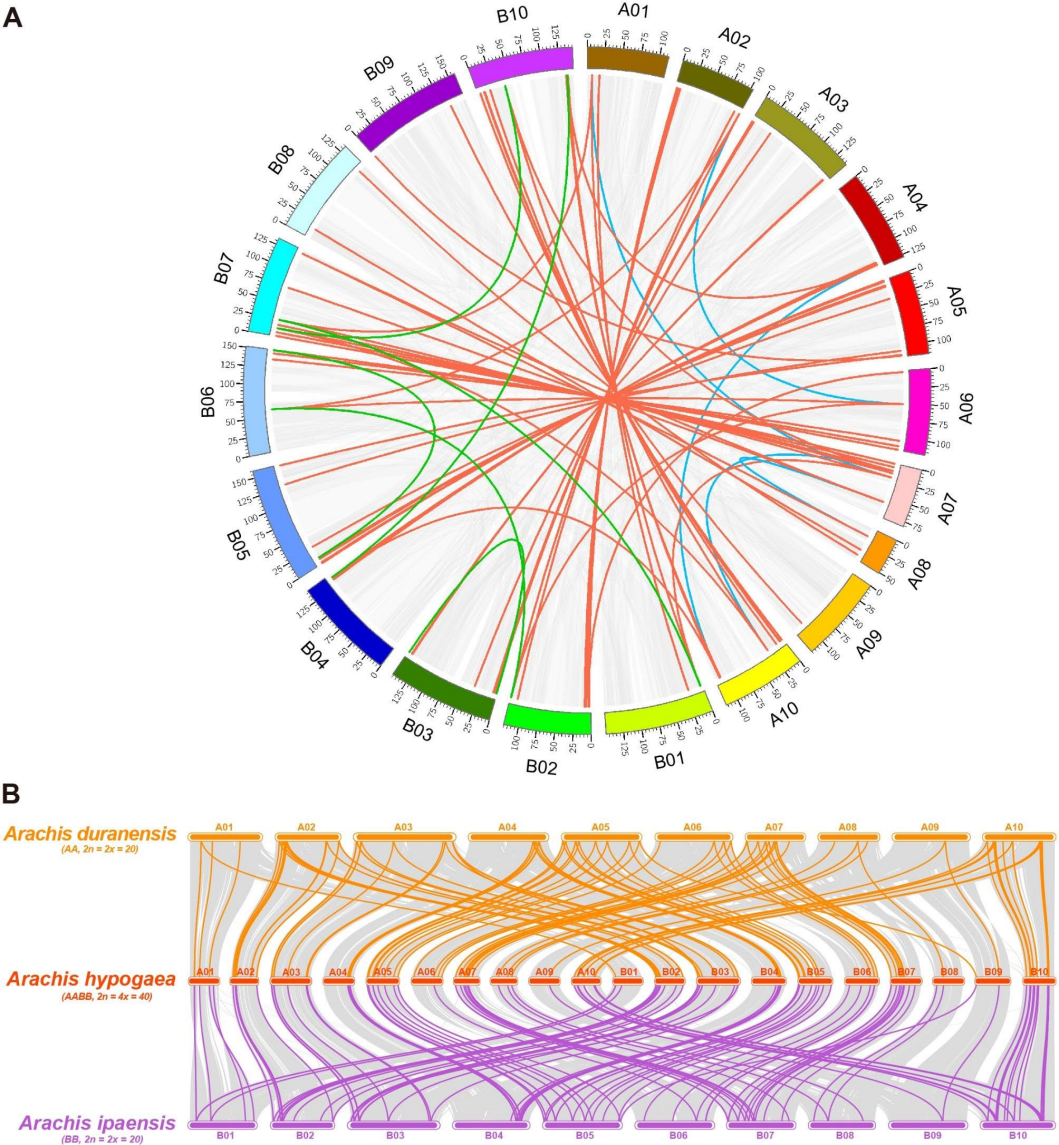
Collinearity relationship of *AhUGT* duplicated gene pairs in *A. hypogaea* and its two diploid ancestors. **A** Synteny analysis of the *AhUGT* genes within *A. hypogaea*. Chromosomes are shown in the outer circle, and indicated by different colors. The red lines indicate the *AhUGT* gene duplications happened between A and B genome, blue lines connect duplicated *AhUGTs* within A genome, and green lines represent duplicated *AhUGTs* within B genome. **B** The collinear blocks of *UGT* genes between *A. hypogaea* and its two diploid ancestors (*A. duranensis*, *A. ipaensis*). Gray lines in the background indicate all collinear blocks, while orange or purple lines highlight collinear *UGT* gene pairs between the two species

Gene duplication event (including tandem and/or segmental duplication) was considered as one of important driven forces for plant gene expansion and evolution (Qiao et al., 2019). As a result, 62 pairs of *AhUGTs* were found to be originated from tandem duplication, some *UGT* gene pairs located closely with each other to generate paralogous clusters on the chromosomes (Fig. 2, Table S6). For example, a gene cluster containing five *AhUGTs* (*AhUGT43*-*AhUGT47*) was observed with tandem duplication on A04, suggesting there were hot spots for gene duplication within this region. In addition, more segmental duplication events (99 pairs of *AhUGT* genes) were identified by collinearity analysis (Fig. 3A, Table S6), indicating segmental duplication might act as one of the main driving forces for the large-scale expansion of *AhUGT* genes. To further investigate selection pressure on the evolution of *AhUGTs*, the ratio of Ka and Ks for duplicated gene pairs was calculated. As a result, the Ka/Ks values of 61 tandem (98.39%) and 91 segmental duplicated pairs (91.92%) were less than 1, indicating that majority of *AhUGTs* underwent strong purifying selection during evolution, leading to limited functional divergence. In contrast, only one tandem (*AhUGT67B1*/*AhUGT67B2*) and two segmental duplicated pairs (*AhUGT16A*/*AhUGT131*, *AhUGT31A*/*AhUGT31B*) had Ka/Ks ratios greater than 1, suggesting they might have undergone positive selection with relatively rapid evolution after duplication. The tandem duplication time of *AhUGTs* was estimated to be occurred between 0.54 and 132.02 million years ago (MYA), while the segmental duplication showed a tentative divergence time from 0.79 to 175.75 MYA.

According to previous genome sequencing, biogeographic and cytogenetic data, *Arachis duranensis* (AA genome, 2n = 2x = 20) and *Arachis ipaensis* (BB genome, 2n = 2x = 20) are the two diploid ancestors of allotetraploid *A. hypogaea* (AABB genome, 2n = 4x = 40) after undergoing a series of multiple hybridizations and replications [37, 50, 51]. To understand the evolutionary process of UGTs from *A. hypogaea* and its two putative ancestral diploids (*A. duranensis* and *A. ipaensis*), the syntenic and collinear relationship maps of orthologous *UGT* pairs were constructed. A total of 119 and 138 *UGT* genes were identified in *A. duranensis* and *A. ipaensis*, respectively (Table S7). Orthologous analysis of *UGT* genes from three peanut species showed that *A. hypogaea* (AABB) had 150 and 149 orthologous *UGT* pairs with *A. duranensis* (AA genome) and *A. ipaensis* (BB genome), respectively (Fig. 3B, Table S8-9). The *UGT* orthologs from the three *Arachis* species displayed similar collinearity with each other. For instance, chromosomes A10, A04, and A02 from *A. duranensis* contained a higher number of collinear genes with *A. hypogaea*. Also, B10, B04 and B02 from *A. ipaensis* contributed the greatest number of collinear genes to *A. hypogaea*. To further explore the collinearity of the peanut *UGT* gene family across model plants, we constructed syntenic maps of *AhUGTs* with those in *A. thaliana*, *G. max* and *G. hirsutum* (Fig. S2). Eventually, *AhUGT* genes exhibited different numbers of collinear lines with the three species, and more orthologous gene pairs were observed in soybean (81) than those in *Arabidopsis* (21) and cotton (66) (Table S10-12). These collinear *AhUGTs* were located unevenly on the 20 chromosomes of *A. hypogaea*, with B02 and B10 harboring more collinear *AhUGT* genes than other chromosomes.

### Analysis of *cis‑* acting elements and transcript expression pattern of *AhUGTs*

A *cis-*acting element is a sequence existed in the flanking sequence of a gene, and is participated in the regulation of gene expression. To investigate the regulatory mechanism, eighteen different types of *cis-*acting elements were characterized in the 1.5 kb upstream region of *AhUGT* genes (Fig. S3, Table S13). According to their functions, these *cis*-acting elements were classified into four major categories, including plant hormone, stress responsiveness, growth and development, and light responsive elements (Fig. S4, Table S14). Ten types of *cis-*acting elements were related to plant hormone category. Among of them, ABRE (ABA responsiveness), TGACG-motif (MeJA responsiveness), and TCA-element (SA responsiveness) were the most abundant plant hormone-responsive elements, which were present in 66.29%, 50.94%, and 32.96% of the *AhUGT* genes, respectively. There were four types elements in the stress responsiveness category, including TC-rich repeats (defense and stress responsiveness), LTR (low-temperature responsiveness), MBS (drought inducibility), and MBSI (regulation of flavonoid metabolism), which accounted for 33.71%, 25.84%, 22.47% and 4.12% of the *AhUGTs*, respectively. In addition, CAT-box (meristem expression) and GCN4_motif (endosperm expression) were found in the growth and development category, with CAT-box (28.46%) accounting for a higher percentage than GCN4_motif (13.11%). Also, two *cis-*elements (G-box, MRE) were observed in the light responsiveness category, with G-box being present in 64.04% of *AhUGTs*, suggesting light signals might have a significant impact on the transcriptional regulation of *AhUGT* expression.

Since gene expression is closely related to *cis-*acting elements, tissue-specific expression patterns of *AhUGTs* were examined in RNA-seq data downloaded from peanutbase, including 22 samples from leaves, roots, shoots, flowers, pegs, and seeds of different maturation stages. It was found that 204 (76%) *AhUGTs* were expressed (FPKM value > 1) in at least one sample (Table S15). Heatmap showed that *AhUGTs* within the same group exhibited distinct expression patterns in different tissues and developmental stages despite they had similar protein motifs (Fig. S5).

**Fig. 4:**
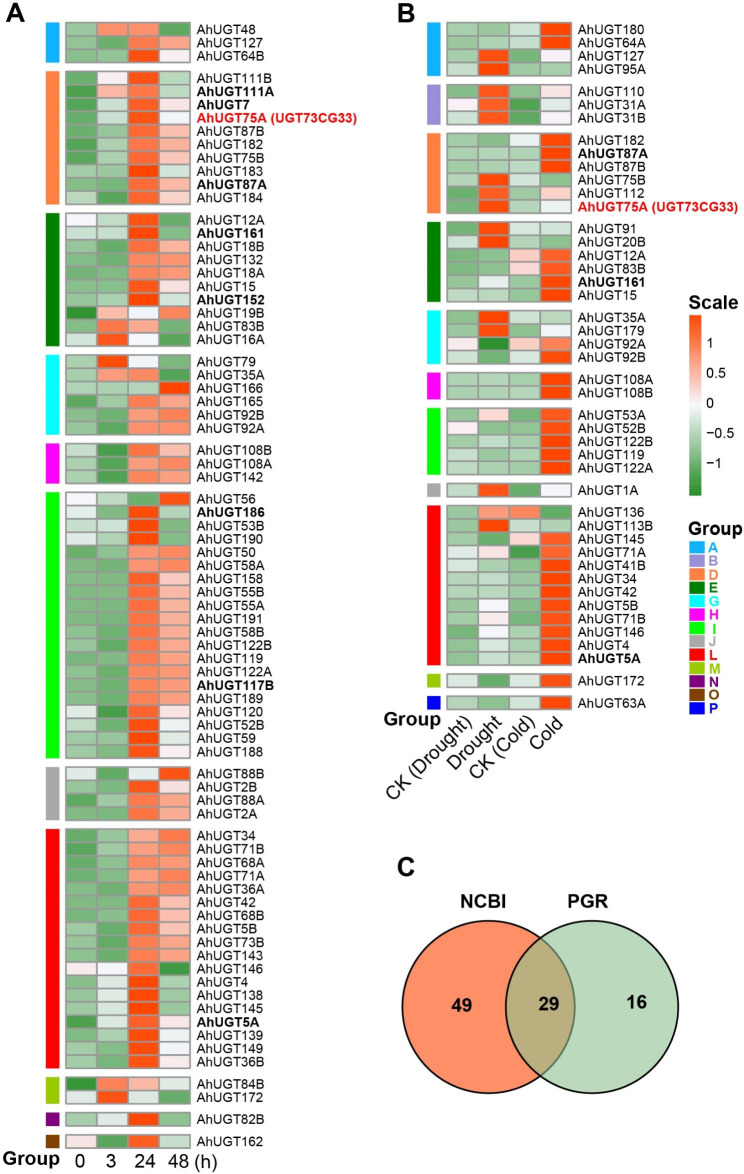
Transcriptomic analysis of *AhUGTs* in response to abiotic stress. **A** Expression patterns of 78 cold-induced *AhUGTs* in leaves after cold treatment at 0, 3, 24, 48 h. The data were download from NCBI database. **B** Expression profiles of 45 up-regulated *AhUGT* transcripts under cold or drought stress. The data were derived from Peanut Genome Resource database. CK indicated control seedlings grown in the normal condition. **C** Venn diagram shows 29 common up-regulated *AhUGTs* in response to drought or cold stress derived from the two transcriptome results. The *AhUGTs* were classified into 16 different groups with different color based on phylogenetic tree. FPKM values were obtained from the RNA transcriptome, and the expression levels of *AhUGTs* were normalized by log_2_ (FPKM + 1) to performed heatmap analysis. The red color in the boxes indicates high expression level, while green represents low level in the heatmap

**Fig. 5:**
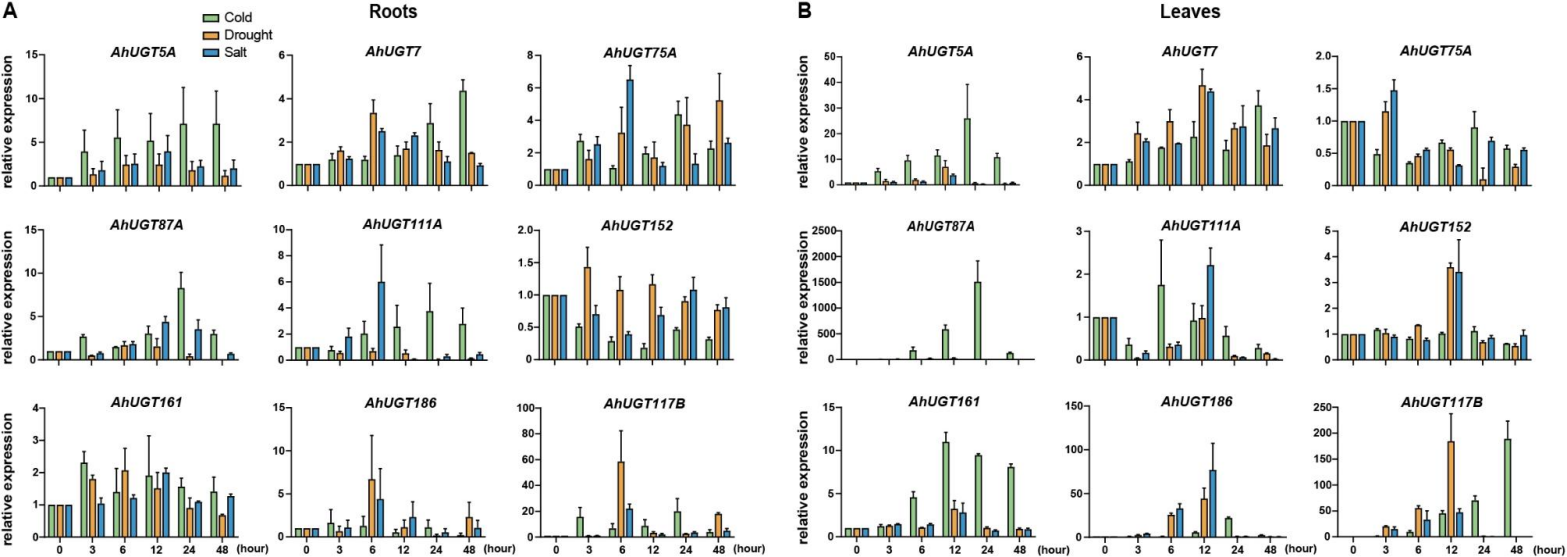
qRT-PCR analysis of transcript levels of *AhUGTs* in roots (**A**) and leaves (**B**) of peanut seedlings under abiotic stress. No treatment control (0 h) was normalized as “1”. Error bars indicate the standard error of three biological replicates

To investigate the transcriptional response of *AhUGT* to abiotic stress, the RNA-seq data were downloaded from Peanut Genome Resource database. A total of 45 *AhUGT* transcripts were significantly induced either by drought or by cold stress (Fig. 4B, Table S16). Additionally, according to our previous transcriptome data [42], the expression levels of 78 *AhUGTs* were enhanced in at least one test time point (3, 24, 48 h) after cold treatment (Fig. 4A, Table S17). Based on the two transcriptome results, there were 29 common up-regulated *AhUGTs* in response to drought or cold stress (Fig. 4C, Table S18), most of them belong to group D, E, I and L. To validate the reliability of transcriptome data, qRT-PCR analysis was used to detected the transcript level of nine selected *AhUGTs* in the roots and leaves of peanut seedlings under abiotic stress (cold, salt, drought). The results showed that most of the genes were up-regulated in response to cold, drought or salt stress, although their expression profiles in the roots and leaves appeared distinct (Fig. 5). The expression profiles of most *AhUGTs* (except *AhUGT152*) correlated well between qRT-PCR results and transcriptome data (Fig. S6), confirming the reliability of transcriptome.

### Biochemical characterization and subcellular localization analysis of *AhUGT75A*

**Fig. 6:**
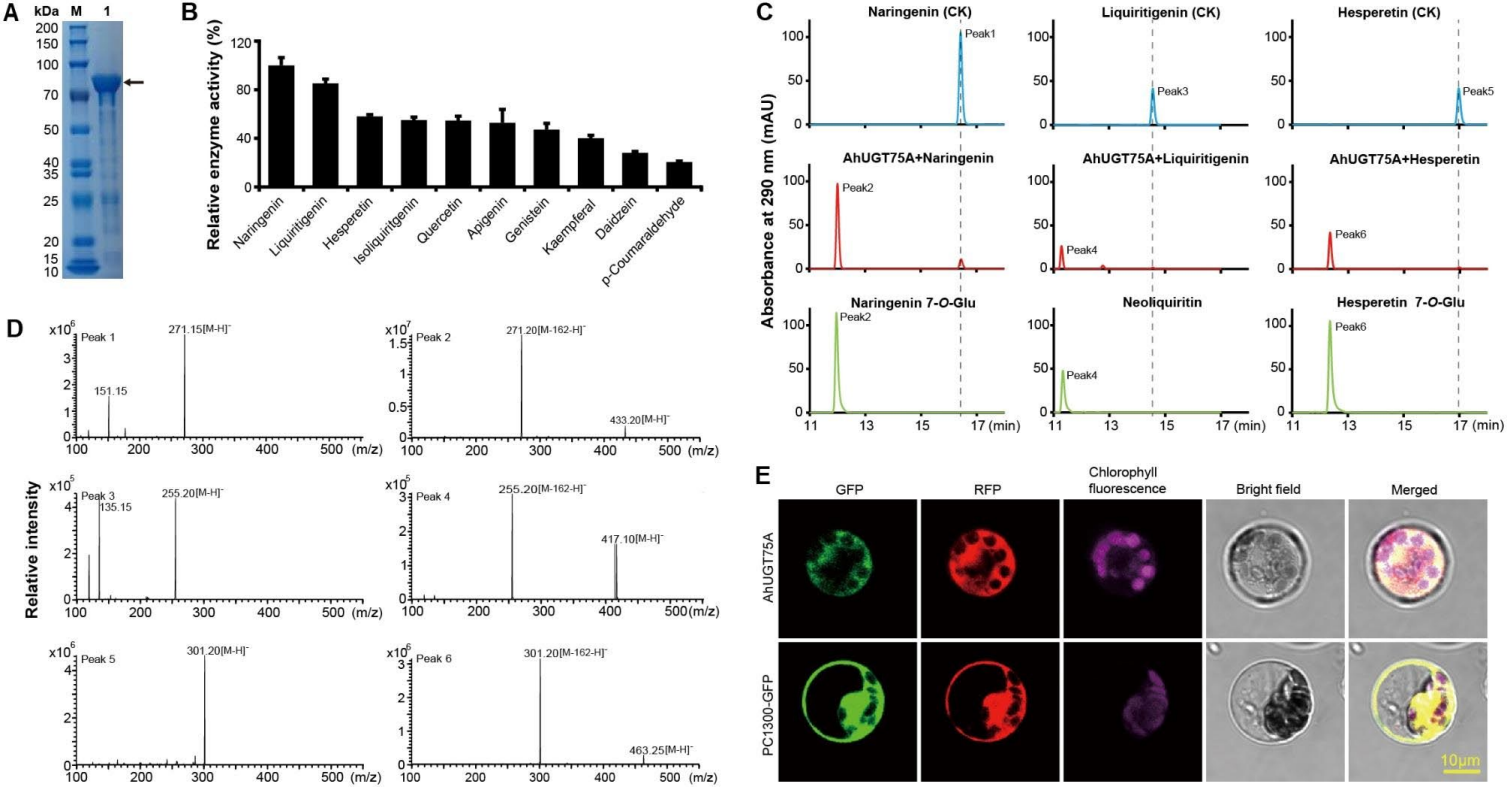
Enzymatic assay and subcellular localization of AhUGT75A. **A** SDS-PAGE analysis of purified recombinant AhUGT75A. The original uncropped image of protein gel electrophoresis was shown in Fig. S8. **B** In vitro enzyme activities of recombinant AhUGT75A toward various flavonoids with the presence of UDP-glucose. **C** HPLC analysis of the products from the reaction catalyzed by recombinant AhUGT75A using naringenin, liquiritigenin or hesperetin as substrate. CK was negative control reaction without protein. **D** Mass spectrum identification of glucosylation products of enzyme reactions. **E** Subcellular localization analysis of AhUGT75A. PC1300-35S::*AhUGT75A*-GFP or PC1300-35S::GFP empty vector was transformed into *Arabidopsis* protoplasts expressing a red fluorescent mitochondrion marker (mitochondrion-RFP) using PEG-mediated method, respectively

Group D was one of major phylogenetic UGT groups in peanut and model plant species. *UGT73* subfamily members within this group were reported to be involved in glycosylation of a board range of substrates, including flavonoids and/or terpenes (Table S19) [52, 53]. *AhUGT75A* was phylogenetically clustered into group D, and encoded a peptide of 496 amino acids with a putative molecular weight of 55.9 KDa. It was designated as *UGT73CG33* by the UGT Nomenclature Committee. According to the tree, *AhUGT75A* was adjacent to soybean *UGT73C19* and *UGT73C20* (Fig. S7), which exhibited glycosylation activities over diverse flavonoid compounds [54, 55]. For biochemical characterization of *AhUGT75A*, it was expressed in *E. coli*, then purified from the bacterial protein extracts via GST affinity chromatography, and finally confirmed by SDS-PAGE (Fig. 6A, Fig. S8). The molecular mass of purified recombinant AhUGT75A protein was approximately 82 kDa, which agreed with the theoretically predicted molecular mass of AhUGT75A plus a GST-tag. In vitro enzyme assays indicated AhUGT75A showed activities toward different flavonoid substrates in presence of UDP-glucose as the sugar donor (Fig. 6B). The best acceptor for AhUGT75A was naringenin, followed by liquiritigenin and hesperetin. LC-MS analysis of the enzyme reaction products showed that AhUGT75A was able to converted the substrates to generate their corresponding 7-*O-*glucosides (e.g. naringenin 7-*O-*glucosides, neoliquiritin and hesperetin 7-*O-*glucoside), confirming it was a flavonoid 7-*O-*UGT (Fig. 6C-D). Next, the kinetic parameters of AhUGT75A toward naringenin and liquiritigenin were measured with UDP-glucose as the sugar donor. The K_m_ values of naringenin and liquiritigenin were 73.0 ± 8.0 μM, and 72.5 ± 9.0 μM, respectively. The K_cat_/K_m_ values of AhUGT75A for naringenin and liquiritigenin were similar (Table 1).

**Table 1:** Analysis of the kinetic parameters of recombinant AhUGT75A protein

Substrates	V_max_ (pKat mg^− 1^)	K_cat_ (s^− 1^)	K_m_ (μM)	K_cat_/K_m_ (M^− 1^s^− 1^)
Naringenin	2183.0 ± 79.4	0.180 ± 0.007	73.0 ± 8.0	2458.3
Liquiritigenin	2254 ± 9 4.03	0.185 ± 0.008	72.56 ± 9.08	2554.7

To determine subcellular location, AhUGT75A-GFP fusion protein was transiently expressed in *Arabidopsis* protoplasts. The green fluorescence signal of AhUGT75A fusion protein was localized to mitochondria, which overlapped with the red fluorescence signal of mitochondria marker, whereas the control GFP protein was distributed throughout the cell (Fig. 6E). These results indicated that AhUGT75A localized to mitochondria.

### Overexpression of *AhUGT75A* confers enhanced abiotic stress tolerance in *Arabidopsis*

**Fig. 7:**
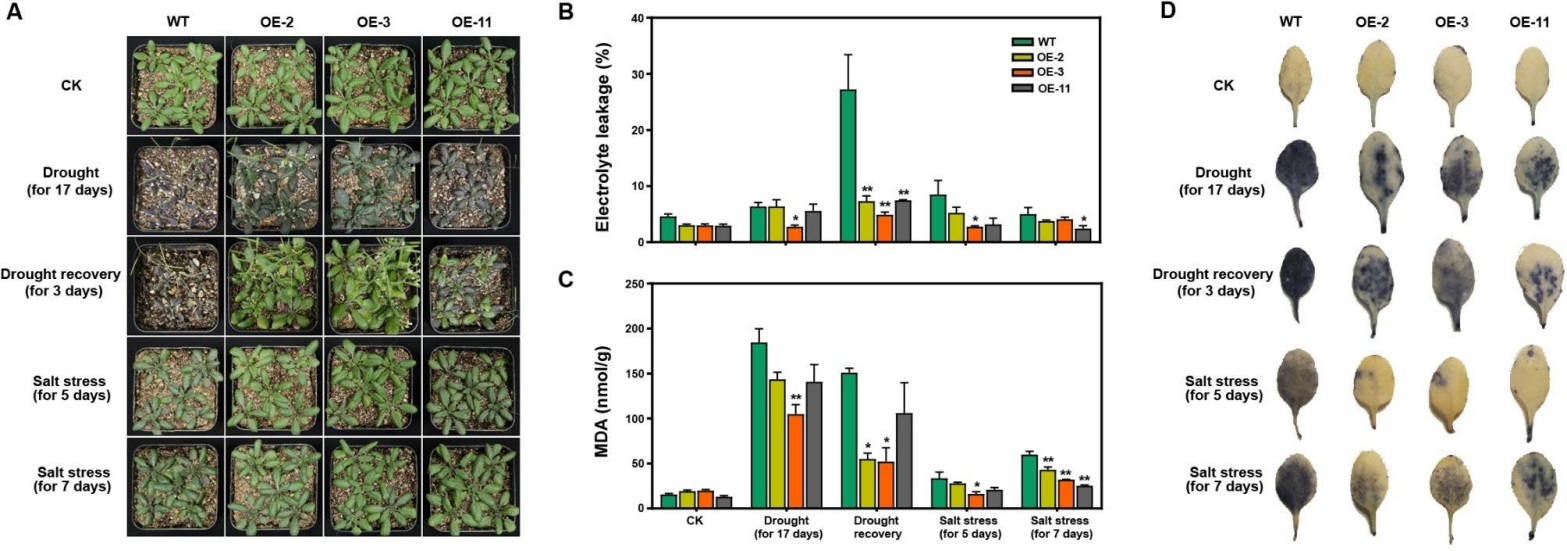
Overexpression of *AhUGT75A* in *Arabidopsis* confers enhanced tolerance against abiotic stress. The growth phenotypes (**A**), electrolyte leakage (**B**), malondialdehyde (MDA) content (**C**) and nitrobluetetrazolium (NBT) staining (**D**) of wild type (WT) and three overexpressing lines under drought and salt stress conditions. Three-week-old seedlings were subjected for stress treatments. For drought stress, the plants were left without watering for 17 days, then re-irrigation for recovery for 3 days. For salt stress, the plants were irrigated with 200 mM NaCl solution instead of water for every 3 days, then the performances of the plants were photographed and the leaves were harvested at five and seven days after salt stress treatment. Plants grown under normal condition (CK) were used as control. Three biological replications for each experiment were taken, one-way ANOVA was used to determine the mean comparison with ± SEM (**P < 0.01, *P < 0.05). Error bars indicate the standard error of three biological replicates

The transcript level of *AhUGT75A* was significantly up-regulated in the roots of the seedlings under cold, salt and/or drought stress (Fig. 5A), indicating it participate in peanut abiotic stress response. To obtain insights into the physiological roles of *AhUGT75A*, we overexpressed the *AhUGT75A* ORF in *Arabidopsis*. Three independent transgenic lines with differing *AhUGT75A* transcript levels were selected for stress tolerance analysis (Fig. S9). When they were exposed to drought and/or salt stress treatments, the overexpressing plants showed better growth performance, less electrolyte leakage, and lower MDA contents compared with wild type (WT), to varying degrees (Fig. 7A-C). Moreover, NBT staining was performed for detecting superoxide level. The three overexpressing lines exhibited lighter and narrow staining than those of the WT plants under stressful conditions, indicating that they accumulated less contents of superoxide (Fig. 7D). These results suggest that *AhUGT75A* might be involved in ROS scavenging, and thus enhanced tolerance to abiotic stress.

## Discussion

### Significant expansion of *AhUGTs* in peanut genome

The UGT multigene family has been profiled in a variety of plant species, such as *Arabidopsis* [56], wheat [57], grape [58] and peach [59]. In current study, a systematic analysis of the UGT family was conducted in peanut, and 267 *AhUGT* genes have been identified. The number of peanut UGTs was more than that in many other higher plants, including rice (182) [8], maize (147) [9], and soybean (149) [10], but less than that in *M. sativa* (409) [11]. The relative expansion of *AhUGT* gene family may be related to the recent whole genome-wide duplication events, as well as many segmental and tandem duplications during evolution in allopolyploid *A. hypogaea*. According to phylogenetic tree, *AhUGT* gene family contained 15 groups (Fig. 1), including 13 groups in *Arabidopsis* (with absence of group C) and two newly discovered group O and P. The lack of group C was also observed in *M. truncatula* and other plants [8]. Previous studies indicated that group E was the largest group in most plant species, including *Arabidopsis* (25), rice (36) (Table S4). However, group I has greatly expanded to contain the largest number of *UGT* genes (53 *AhUGTs*) in peanut, accounting for 19.9% of total putative *UGTs*. Similar results were found in another legume *M. sativa* [11]. Gene duplication is important for gene family expansion, and might give rise to gene clusters on chromosomes. In this study, about 70% *AhUGT* genes in group I underwent either tandem or segmental duplication events. It should be noted that *AhUGTs* from this group were found to form large gene clusters and located specifically on the chromosomes A04, B04, A10, and B10. As previously reported, rice *UGT83A1* (GSA1), belonging to group I, was demonstrated to be induced by abiotic stresses, and catalyze flavonoid glycosylation to enhance plant tolerance [60]. Also, overexpression of an apple *MdUGT83L3* (*Md09G1064900*) from group I increased anthocyanin accumulation in callus tissue and improved ROS scavenging capacity under salt and cold stress [61]. According to our transcriptome data, about half of *AhUGTs* in group I was up-regulated by cold and/or drought stress (Fig. 4). qRT-PCR analysis also confirmed that the transcript levels of *AhUGT186* and *AhUGT117B* were significantly increased under cold, salt and/or drought stress both in leaves and roots of the seedlings. These findings suggested significant expansion of group I would facilitate the evolution of peanut in adapting to different environmental stress conditions.

### *AhUGTs* are involved in peanut growth and abiotic stress response

Plant UGTs play an essential role in regulating growth and development. To explore the function of *AhUGTs* in peanut, the transcriptional patterns in various tissues and during different developmental stages were analyzed based on the online RNA-seq data. It was found that 204 (76%) *AhUGT* genes were expressed in at least one tissue. Similar patterns were discovered in maize and *Linum usitatissimum*, wherein 82% and 73% of *UGT* genes were found to be expressed in at least one tissue [9, 62]. Notably, three *AhUGTs* belonging to group E (*AhUGT83A*, *AhUGT83B* and *AhUGT176*) showed constitutive high expression levels (with FPKM value more than 10) in all the 22 sample tissues, indicating they might be involved in growth and development of peanut. Accumulated genetic evidences have suggested that UGT families can broadly participate in the plant response to abiotic stresses [63]. ABA acts as a major signaling molecule involved in response to drought stress through regulating developmental and physiological processes in crops, including stomata closure. In this study, 177 *AhUGT* genes were found to contain ABA responsiveness *cis-*acting elements (ABRE) in their promoter regions, 90 *AhUGTs* possess TC-rich repeat elements that are involved in defense and stress responsiveness, and 69 genes have low-temperature responsiveness (LTR) elements. Taken together, nearly 80% of *AhUGT* genes contained at least one type of stress responsiveness *cis-*acting elements (ABRE, LTR, TC-rich), suggesting their potential involvements in abiotic stress response. Accordingly, a total of 94 *AhUGT* transcripts were significantly induced either by drought or by low temperature stress based on the RNA-seq results (Table S16-17), most of which were found to have ABRE, LTR or TC-rich *cis*-acting elements in their promoter regions, indicating that *AhUGTs* were broadly involved in abiotic stress response in peanut.

### *AhUGT75A* plays important roles in abiotic stress tolerance through ROS scavenging

During last two decades, many UGTs have been extensively studied regarding their expression profiles, enzyme activities, and biological roles in various plant species. *UGT73* belonging to the group D was one of well-characterized family, whose members displayed broad activities toward diverse substrates, including terpenoids [52], flavonoids [53], and brassinosteroids [64]. Given the rich sources of flavonoids in peanuts, it was proposed that many flavonoid-modifying *UGTs* might exist in the peanut genome. In present study, we identified *AhUGT75A* (*UGT73CG33*), a stress-induced peanut *UGT* belonging to *UGT73* family in group D. Like soybean *UGT73C19* and *UGT73C20* [54, 55], *AhUGT75A* was characterized as a flavonoid 7-*O-*UGT by in vitro enzyme assays. Also, the transcript level of *AhUGT75A* was strongly induced by abiotic stress. Overexpression of *AhUGT75A* in *Arabidopsis* enhance tolerance against drought and salt stress. The level of lipid peroxidation was evaluated by measuring MDA content. The lower accumulation of MDA and lighter NBT staining in the overexpressing lines suggested that they were suffered from less oxidative damage caused by drought or salt stress. Similarly, the *GmUGT73F4* transgenic *Arabidopsis* plants showed higher antioxidant enzyme activities (superoxide dismutase, catalase, peroxidase), and less content of MDA and H_2_O_2_ than the WT plants when exposed to high temperature treatments [24]. As a homologous protein of soybean *GmUGT73F2*, *GmUGT73F4* was reported to exhibited glycosylation activities towards the flavonoids that involved in antioxidant and scavenging oxygen free radicals [65]. Taken together, *UGT73* family members, including *AhUGT75A*, played important roles in enhancing tolerance to abiotic stresses probably through ROS scavenging.

## Conclusions

This study provided comprehensive insights into the phylogenetic relationships, gene structures, duplications, and expression profiling of UDP-glycosyltransferases in *A. hypogaea*. A total of 267 *AhUGTs* were identified in peanut genome. The *AhUGT* genes were distributed unevenly among the 20 chromosomes, and clustered into 15 phylogenetic groups with a significant expansion of group I. Segmental duplication was the major driver for *AhUGT* gene family expansion. Expression profiles analysis indicated *AhUGTs* were involved in peanut growth and abiotic stress response. *AhUGT75A* was induced by abiotic stress and identified as a flavonoid 7-*O-*UGT belonging to *UGT73* subfamily, which played important roles in conferring abiotic stress tolerance through ROS scavenging.

### Electronic supplementary material

Below is the link to the electronic supplementary material.

**Supplementary Material 1: Fig. S1** Gene structure and conserved protein motifs of AhUGT family members. A The phylogenetic tree of AhUGTs. B Exon-intron structure of *AhUGT* genes. Red boxes represent coding sequences, blue boxes are untranslated regions, and black lines indicate introns. C Conserved protein motifs in AhUGTs. **Fig. S2** Syntenic relationship analysis of UGTs between *A. hypogaea* and other plant species. MCScanX program was used to analyze the orthologous genes between *A. hypogaea* and *A. thaliana*, *G. max* and *G. hirsutum*. Gray lines represent all orthologous gene pairs, and color lines highlight UGT orthologous gene pairs. **Fig. S3** Predicted *cis*-acting elements in the promoter regions of *AhUGT* genes. A The phylogenetic tree. B Prediction of *cis*-acting elements in the 1500 bp upstream region of *AhUGT* gene transcription start site. **Fig. S4** Classification and annotation of *cis-*acting elements in the promoter regions of *AhUGTs*. The values on the top of bar indicate the number of *cis*-acting elements. **Fig. S5** Transcriptional expression profiles of *AhUGTs* in various peanut tissues and different developmental stages. The *AhUGTs* were classified into 15 different groups with different color based on phylogenetic tree. FPKM values were obtained from the RNA-seq data, and the expression levels of *AhUGTs* were normalized by log_2_ (FPKM + 1), with red to green indicating high to low gene expression level in the heatmap. **Fig. S6** Analysis of transcriptional patterns of nine selected *AhUGTs* in response to cold stress. The expression levels of *AhUGTs* were analyzed by qRT-PCR (shown by green bars) and transcriptome data (indicated by red lines). No treatment control (0 h) was normalized as “1”. Error bars indicate the standard error of three biological replicates. The expression profiles of most *AhUGTs* (except *AhUGT152*) correlated well between qRT-PCR results and transcriptome data. **Fig. S7** Phylogenetic analysis of AhUGTs from group D. The phylogenetic tree was constructed using the maximum likelihood method by aligning the amino acid sequences of 31 AhUGTs from group D with those of functionally characterized UGT73 subfamily members from other plant species. **Fig. S8** SDS-PAGE analysis of purified recombinant AhUGT75A. This is the original uncropped image of protein gel electrophoresis. The red rectangle region in this image corresponds to Fig. 6A. **Fig. S9** qRT-PCR analysis of relative transcript level of *AhUGT75A* in three overexpressing lines and wild type Arabidopsis plants. Tubulin was used as an internal reference gene. Data were derived from three biological replicates and are presented as means ± SEM

**Supplementary Material 2: Table S1.** Primers used in this study. **Table S2**. Idenfication and physical properties of Family-1 UDP-glycosyltransferases members in *Arachis hypogaea*. **Table S3.** Functionally characterized UGTs from other plant species. **Table S4.** The number of UGTs identified for each phylogenetic group in different plant species. *gymnosperm-divergent groups (G-DVs) *free-sporing plant divergent groups (FS-DVs) *Outgroup (OG). **Table S5.** The relative expansion and contraction ratio of UGT group in different plants. *gymnosperm-divergent groups (G-DVs) *free-sporing plant divergent groups (FS-DVs) *Outgroup (OG). **Table S6.** The Ka/Ks ratios for duplicated *AhUGT* genes in *Arachis hypogaea*. **Table S7**. Idenfication and physical properties of UGT members in two ancestral diploid peanuts. **Table S8**. Orthologous UGT gene pairs between *Arachis hypogaea* and Arachis duranensis. *The size of the colinearity region. **Table S9.** Orthologous UGT gene pairs between *Arachis hypogaea* and *Arachis ipaensis.* *The size of the colinearity region. **Table S10.** Orthologous *UGT* gene pairs between *Arachis hypogaea* and *Glycine max.* *The size of the colinearity region. **Table S11.** Orthologous UGT gene pairs between *Arachis hypogaea* and *Arabidopsis thaliana.* *The size of the colinearity region. **Table S12.** Orthologous UGT gene pairs between *Arachis hypogaea* and *Gossypium hirsutum*. *The size of the colinearity region. **Table S13**. Prediction of *cis*-acting elements in the promoter regions of *AhUGT* genes. **Table S14**. Summary of *cis-*acting elements in the promoter of *AhUGT* genes. **Table S15.** Transcript levels of *AhUGTs* in various tissues and different developmental stages. RNA-seq data were derived from peanutbase website database. The values represent Fragment Per Kilobase Million. **Table S16.** Transcriptional level of *AhUGTs* in response to cold or drought stress. The RNA-data were derived from Peanut Genome Resource. Plants grown under normal condition (CK) were used as control. **Table S17.** Transcriptional level of *AhUGTs* after cold stress at different treatment time point. RNA-seq data were derived from NCBI. **Table S18**. 29 common up-regulated *AhUGTs* in response to drought or cold stress from the two RNA-seq data. **Table S19**. Functionally characterized UGT73 subfamily members in plant species

## Data Availability

All databases used in this study are open for public and the links are as follows: Peanutbase: https://www.peanutbase.org, NCBI: https://www.ncbi.nlm.nih.gov/, Pfam: http://pfam.xfam.org, SMART: http://smart.embl.de, MEME: https://meme-suite.org, FigTree: http://tree.bio.ed.ac.uk/, PlantGDB: http://www.plantgdb.org, PlantCARE: http://bioinformatics.psb.ugent.be/webtools/plantcare/, Peanut Genome Resource: http://peanutgr.fafu.edu.cn, ExPASy: https://prosite.expasy.org, CELLO: http://cello.life.nctu.edu.tw, TAIR: http://www.arabidopsis.org/, CottonGen: https://www.cottongen.org, GSDS: http://gsds.gao-lab.org. The accession numbers of *AhUGTs* are listed in Table S2. All the nucleotide and protein sequences of *AhUGTs* are available on the Peanutbase: https://www.peanutbase.org.

## Abbreviations

Aa: Amino acids
ABA: Abscisic acid
ABRE: Abscisic acid responsiveness element
kDa: Kilo Dalton
LTR: Low temperature response
MBS: MYB binding site
MeJA: Methyl jasmonate
pI: Protein Isoelectric point
qRT-PCR: Quantitative Real-time Polymerase Chain Reaction
SA: Salicylic acid
