# Supplementary material for: Genome-wide analysis of UDP-glycosyltransferase gene family and identification of a flavonoid 7-O-UGT (AhUGT75A) enhancing abiotic stress in peanut (Arachis hypogaea L.)
Source: BMC Plant Biol. 2023 Dec 7;23:626. doi: 10.1186/s12870-023-04656-3 (PMC10702079; doi:10.1186/s12870-023-04656-3)
Supplement: Supplementary file 1 — Supplementary Material 1: Fig. S1 Gene structure and conserved protein motifs of AhUGT family members. A The phylogenetic tree of AhUGTs. B Exon-intron structure of AhUGT genes. Red boxes represent coding sequences, blue boxes are untranslated regions, and black lines indicate introns. C Conserved protein motifs in AhUGTs. Fig. S2 Syntenic relationship analysis of UGTs between A. hypogaea and other plant species. MCScanX program was used to analyze the orthologous genes between A. hypogaea and A. thaliana, G. max and G. hirsutum. Gray lines represent all orthologous gene pairs, and color lines highlight UGT orthologous gene pairs. Fig. S3 Predicted cis-acting elements in the promoter regions of AhUGT genes. A The phylogenetic tree. B Prediction of cis-acting elements in the 1500 bp upstream region of AhUGT gene transcription start site. Fig. S4 Classification and annotation of cis-acting elements in the promoter regions of AhUGTs. The values on the top of bar indicate the number of cis-acting elements. Fig. S5 Transcriptional expression profiles of AhUGTs in various peanut tissues and different developmental stages. The AhUGTs were classified into 15 different groups with different color based on phylogenetic tree. FPKM values were obtained from the RNA-seq data, and the expression levels of AhUGTs were normalized by log2 (FPKM + 1), with red to green indicating high to low gene expression level in the heatmap. Fig. S6 Analysis of transcriptional patterns of nine selected AhUGTs in response to cold stress. The expression levels of AhUGTs were analyzed by qRT-PCR (shown by green bars) and transcriptome data (indicated by red lines). No treatment control (0 h) was normalized as “1”. Error bars indicate the standard error of three biological replicates. The expression profiles of most AhUGTs (except AhUGT152) correlated well between qRT-PCR results and transcriptome data. Fig. S7 Phylogenetic analysis of AhUGTs from group D. The phylogenetic tree was constructed using [file 12870_2023_4656_MOESM1_ESM.pdf]

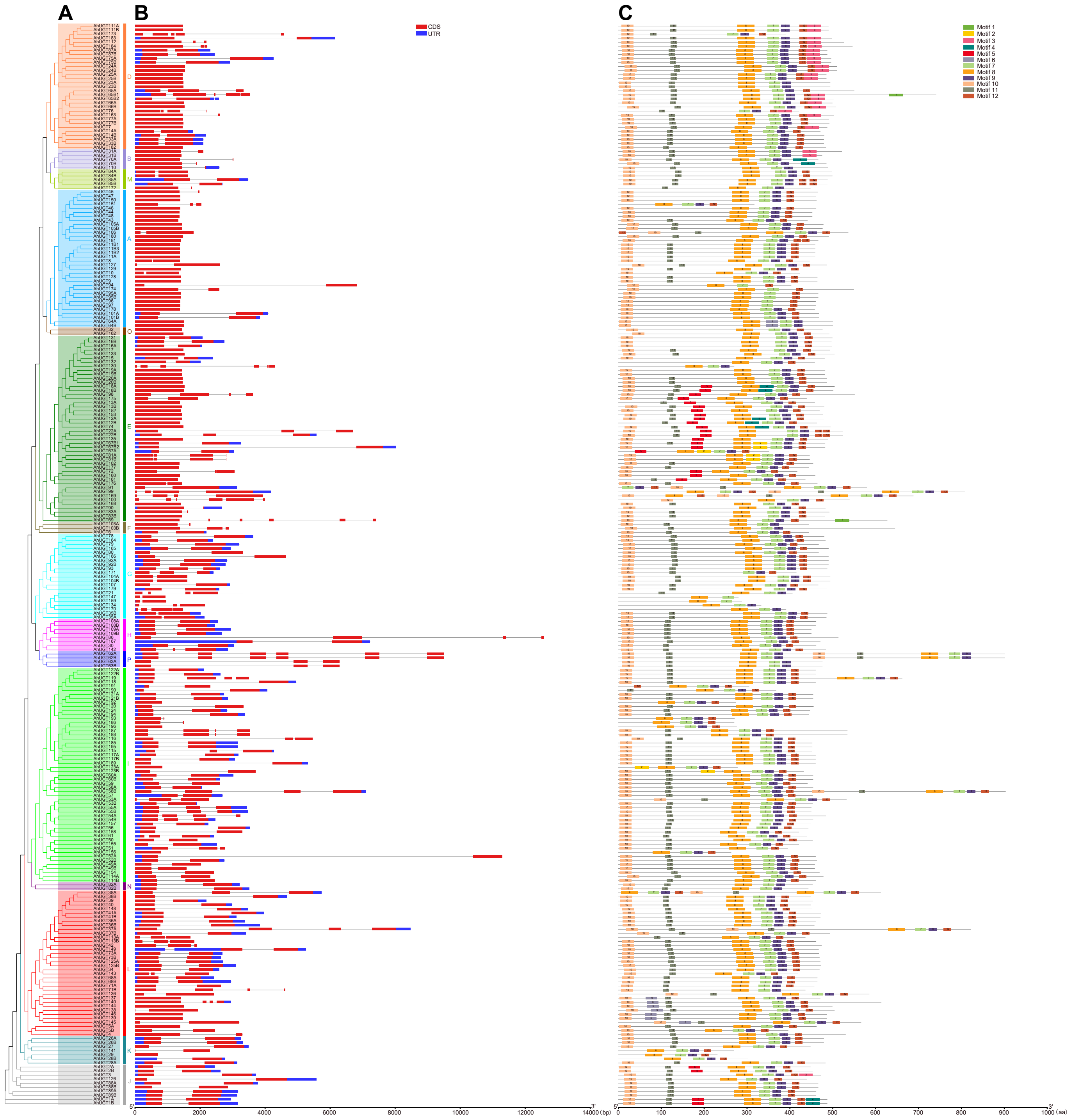

Fig. S1 Gene structure and conserved protein motifs of AhUGT family members. **A** The phylogenetic tree of AhUGTs. **B** Exon-intron structure of *AhUGT* genes. Red boxes represent coding sequences, blue boxes are untranslated regions, and black lines indicate introns. **C** Conserved protein motifs in AhUGTs

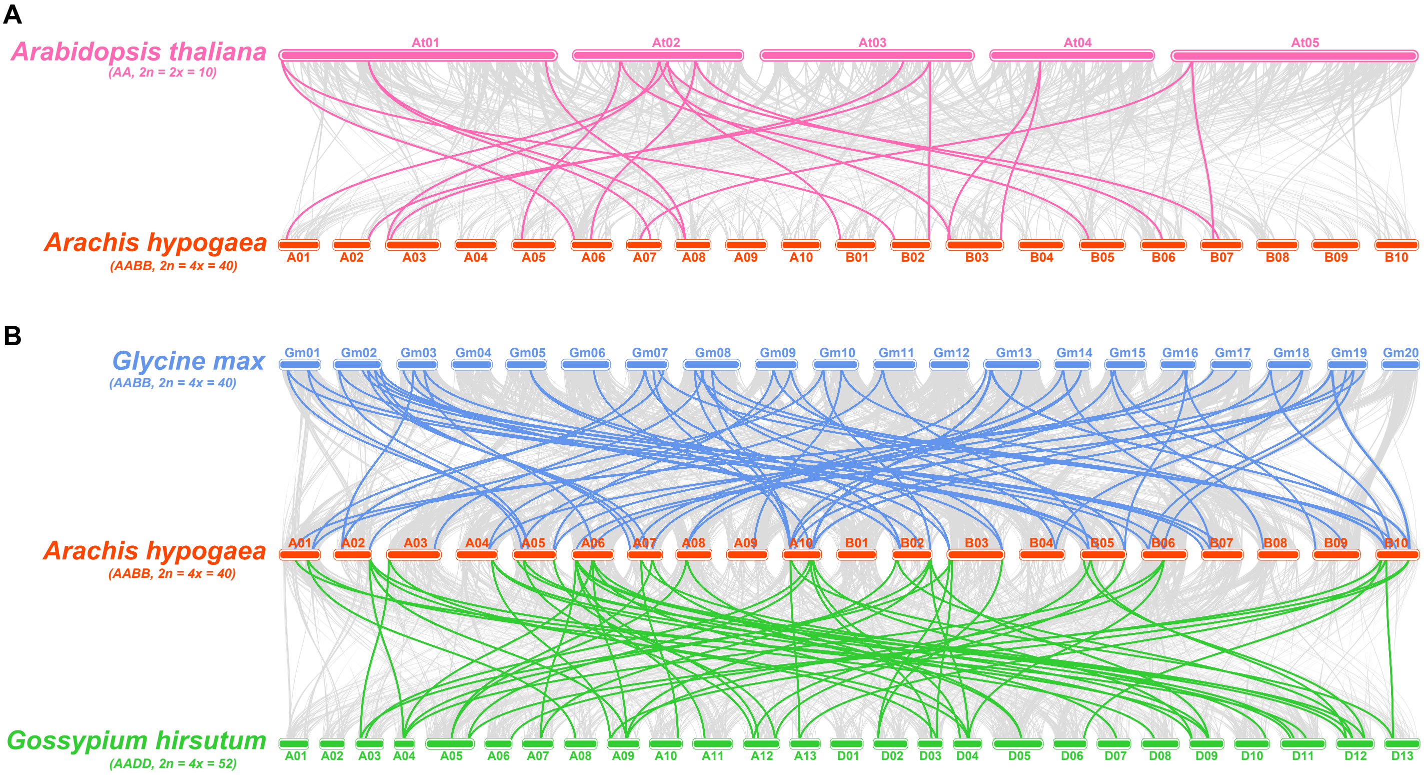

Fig. S2 Syntenic relationship analysis of UGTs between *A. hypogaea* and other plant species. MCScanX program was used to analyze the orthologous genes between *A. hypogaea* and *A. thaliana*, *G. max* and *G. hirsutum*. Gray lines represent all orthologous gene pairs, and color lines highlight UGT orthologous gene pairs

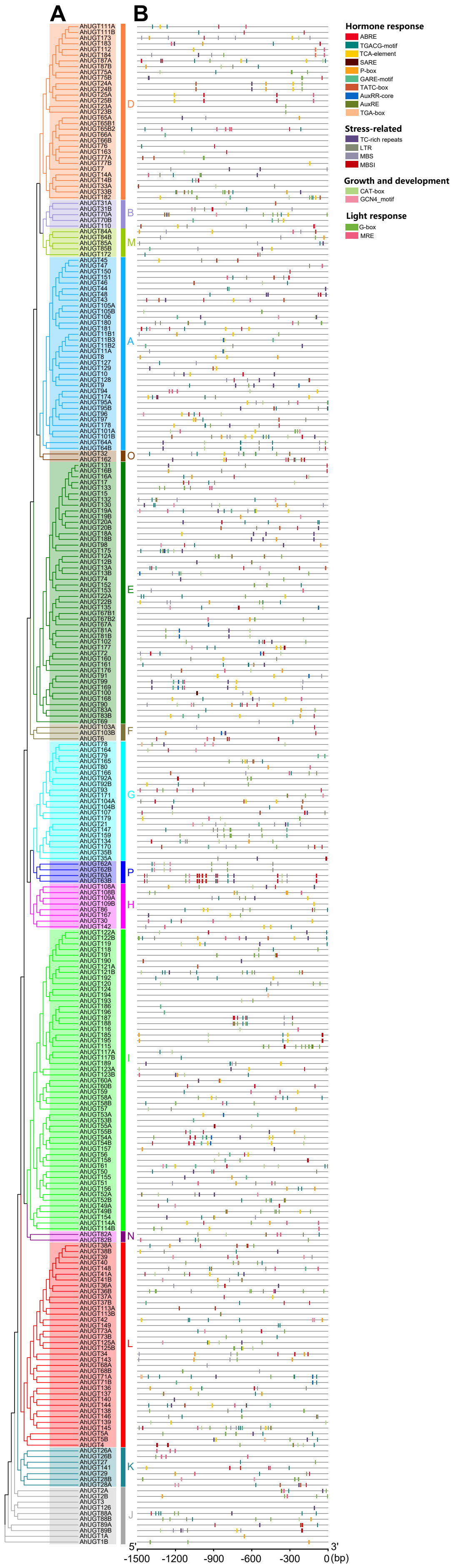

Fig. S3 Predicted *cis*-acting elements in the promoter regions of *AhUGT* genes. A The phylogenetic tree. **B** Prediction of *cis*-acting elements in the 1500 bp upstream region of *AhUGT* gene transcription start site

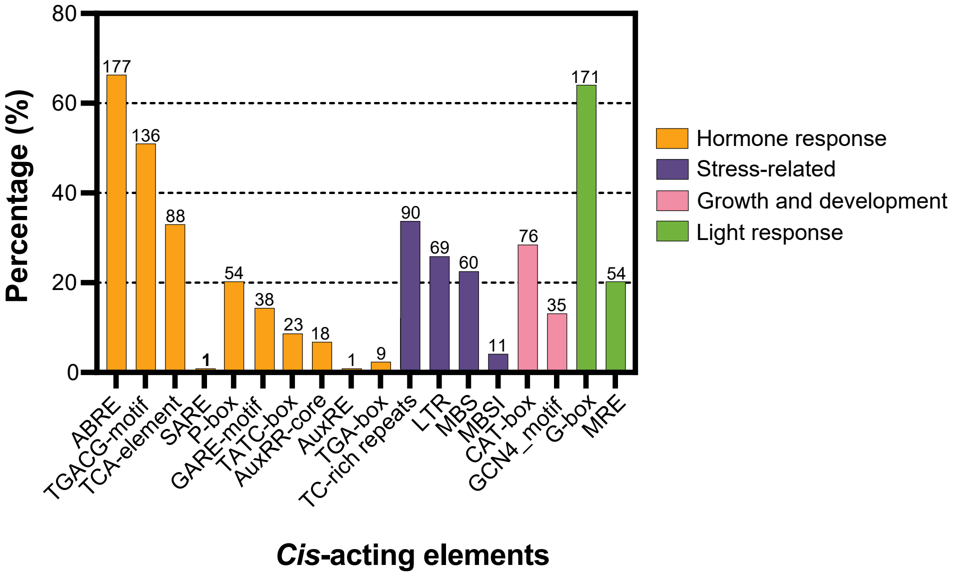

Fig. S4 Classification and annotation of *cis*-acting elements in the promoter regions of *AhUGTs*. The values on the top of bar indicate the number of *cis*-acting elements

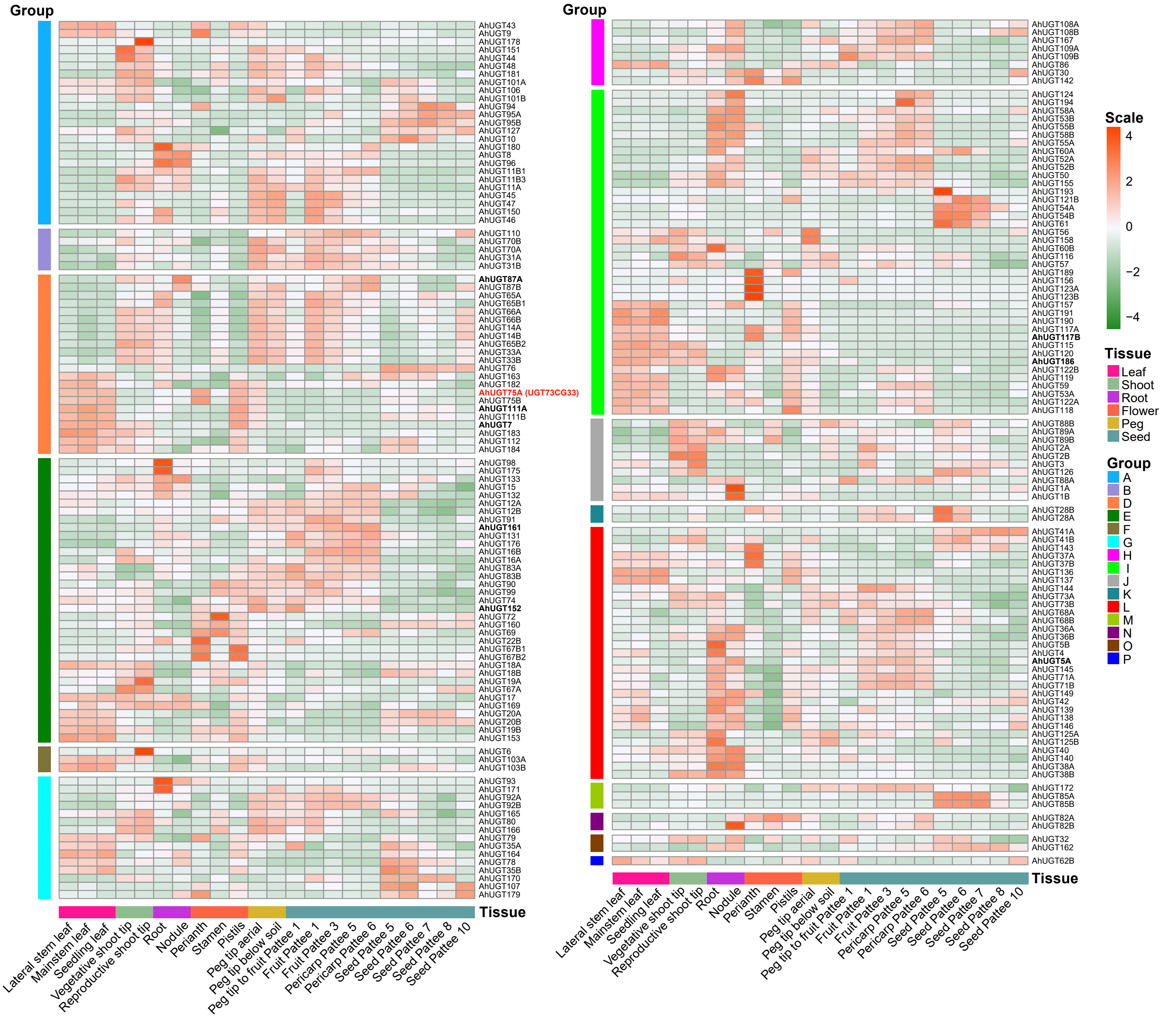

Fig. S5 Transcriptional expression profiles of *AhUGTs* in various peanut tissues and different developmental stages. The *AhUGTs* were classified into 15 different groups with different color based on phylogenetic tree. FPKM values were obtained from the RNA-seq data, and the expression levels of *AhUGTs* were normalized by $\log_2$ (FPKM+1), with red to green indicating high to low gene expression level in the heatmap

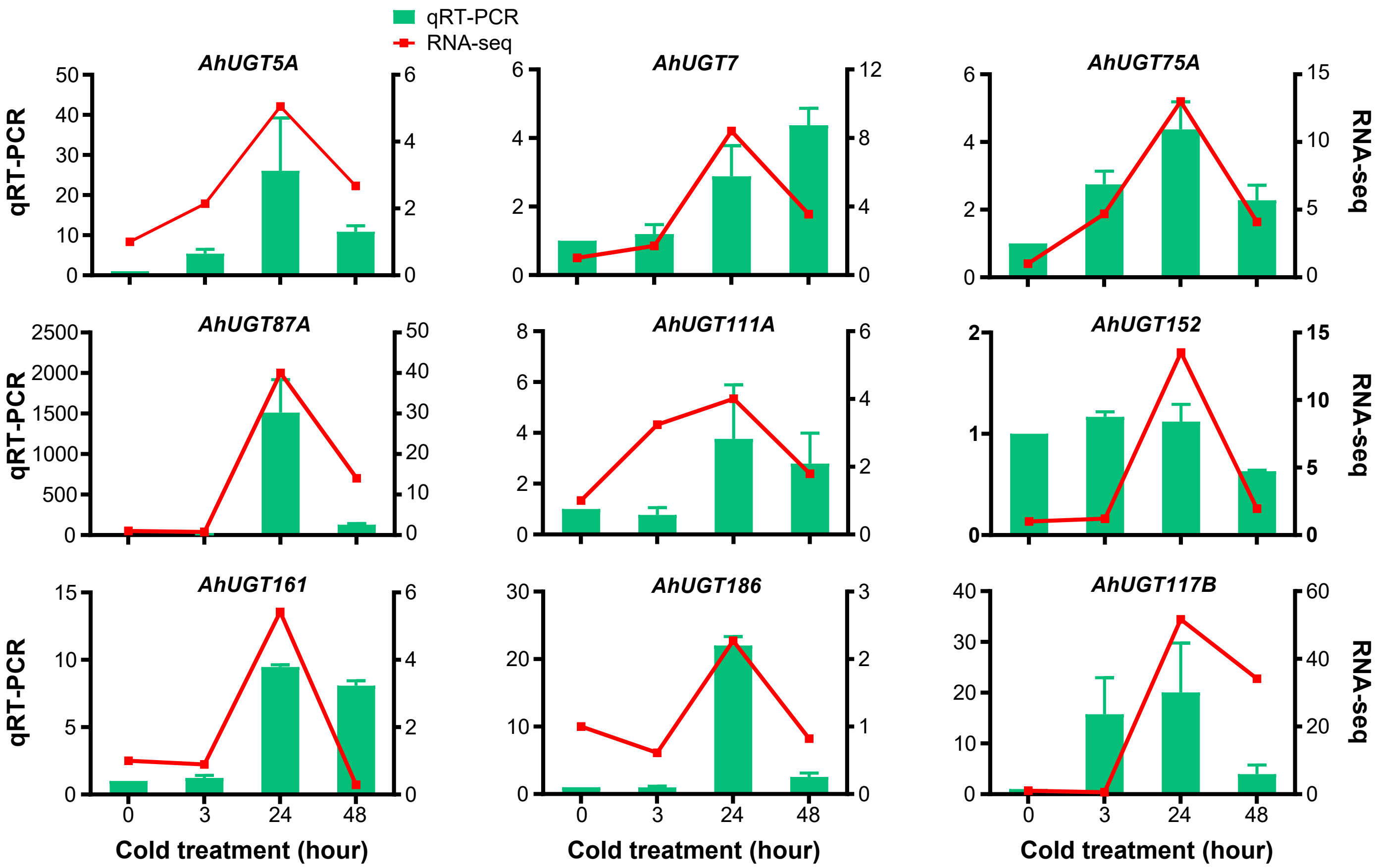

Fig. S6 Analysis of transcriptional patterns of nine selected *AhUGTs* in response to cold stress. The expression levels of *AhUGTs* were analyzed by qRT-PCR (shown by green bars) and transcriptome data (indicated by red lines). No treatment control (0 h) was normalized as "1". Error bars indicate the standard error of three biological replicates. The expression profiles of most *AhUGTs* (except *AhUGT152*) correlated well between qRT-PCR results and transcriptome data

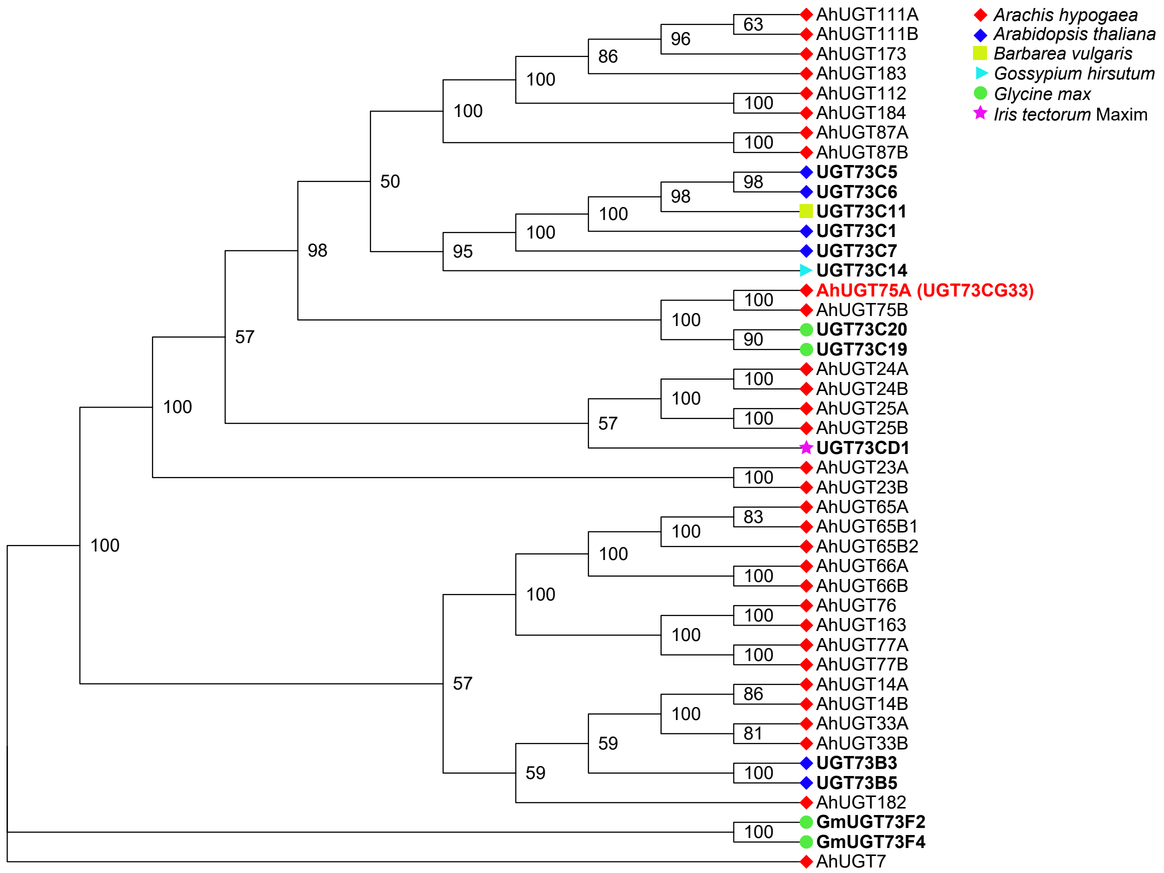

Fig. S7 Phylogenetic analysis of AhUGTs from group D. The phylogenetic tree was constructed using the maximum likelihood method by aligning the amino acid sequences of 31 AhUGTs from group D with those of functionally characterized UGT73 subfamily members from other plant species

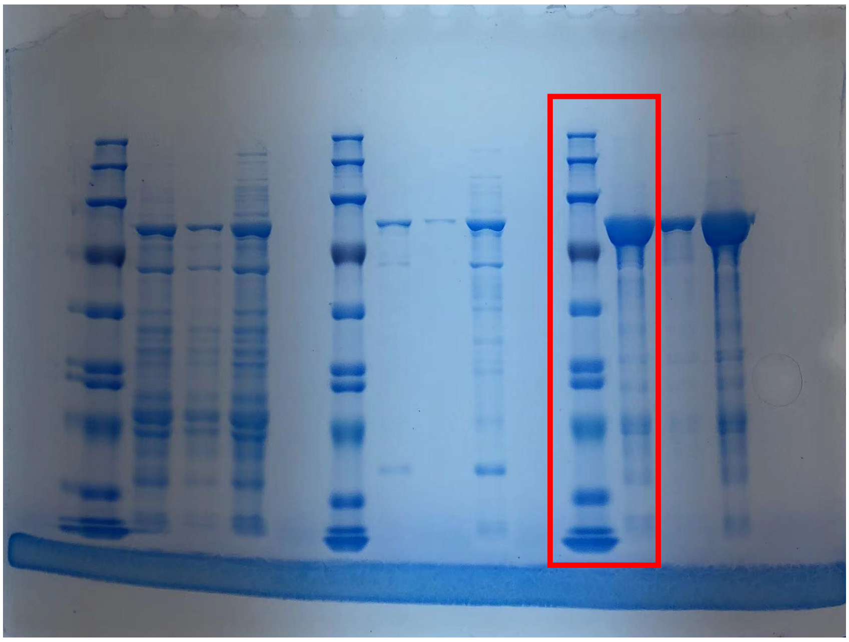

Fig. S8 SDS-PAGE analysis of purified recombinant AhUGT75A. This is the original uncropped image of protein gel electrophoresis. The red rectangle region in this image corresponds to Figure 6A

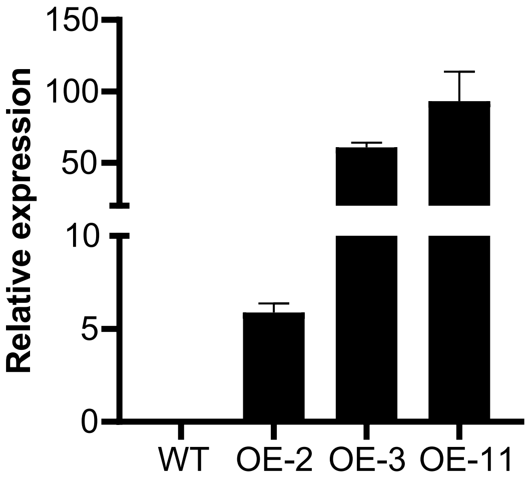

Fig. S9 qRT-PCR analysis of relative transcript level of *AhUGT75A* in three overexpressing lines and wild type Arabidopsis plants. Tubulin was used as an internal reference gene. Data were derived from three biological replicates and are presented as means ± SEM
